# Identification of Differentially Expressed Profiles of Alzheimer's Disease Associated Circular RNAs in a Panax Notoginseng Saponins-Treated Alzheimer's Disease Mouse Model

**DOI:** 10.1016/j.csbj.2018.10.010

**Published:** 2018-11-14

**Authors:** Jin-Lan Huang, Zhe-Hao Xu, Si-Man Yang, Chao Yu, Fan Zhang, Mei-Chun Qin, Yan Zhou, Zhen-Guo Zhong, Deng-Pan Wu

**Affiliations:** a Scientific Research Center of Traditional Chinese Medicine, Guangxi University of Chinese Medicine, Nanning, Guangxi 530200, China; b Jiangsu Key Laboratory of New Drug Research and Clinical Pharmacy, Pharmacy School, Xuzhou Medical University, Xuzhou, Jiangsu 221004, China; c Department of Pharmacology, Pharmacy School, Xuzhou Medical University, Xuzhou, Jiangsu 221004, China

**Keywords:** Alzheimer's disease, Circular RNAs, Expression profiles, Panax notoginseng saponins

## Abstract

Circular RNAs (circRNAs) play vital roles in AD pathogenesis. Thus, developing therapeutic candidates targeting circRNA may provide a novel therapeutic strategy for AD treatment. Our previous studies showed that Panax notoginseng saponins (PNS) could significantly prohibit the pathological progress of AD. However, the mechanisms by which PNS attenuates AD progression is still unclear. The present study shows that PNS may exhibit an ability to modulate the expression of AD-associated circRNAs. Specifically, PNS treatment leads to five circRNAs upregulation and two circRNAs downregulation, indicating that the therapeutic effect of PNS against AD may be associated with its role in the regulation of circRNA expression. Next, mmu_circRNA_013636 and mmu_circRNA_012180 were selected and GO and KEGG analyses were performed to further investigate the biological functions and potential mechanisms of these circRNAs. The results showed that the selected circRNAs were involved in AD-associated biological process and pathways, suggesting that these circRNAs may participate in AD pathogenesis. Collectively, our study indicates that the therapeutic effects of PNS on AD may be through modulating the expression of AD associated circRNAs and suggests that PNS is a potential circRNA-targeted agent against AD, which may provide useful resources for developing potential candidates targeting circRNAs against AD.

## Highlights

• 10 dysregulated circRNAs were found in PNS treated and untreated SAMP8 mice.
• Mmu_circRNA_013636 are related with AD-associated biological processes and pathways.
• Mmu_circRNA_012180 are related with AD-associated biological processes and pathways.
• CircRNAs may be potential biomarkers or therapeutic targets in AD.
• PNS is a potential circRNA-targeted agent against AD.

## Graphical Abstract

**Figure:**
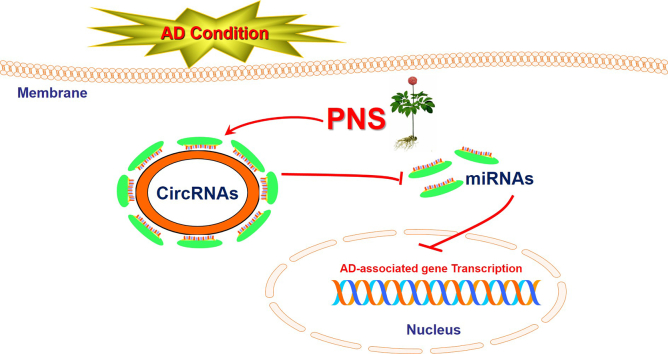

## 1 Introduction

Alzheimer's disease (AD), which is characterized by amyloid-β (Aβ) and neurofibrillary tangles accumulation, neuronal and synaptic loss, and cognitive decline, is becoming one of the most common forms dementia among the elderly. An epidemiologic study showed that there were an approximate 4.76 million individuals aged 65 and older suffering from AD in 2016 and 13.8 million people is expected to develop AD by mid-century in the United States alone [1]. Despite researchers have made efforts to try to explain the pathogenesis of AD, unfortunately, no effective therapeutic strategies can prevent the progression of AD currently. Thus, discovery of novel therapeutic targets is an urgent need and developing potential candidates targeting these potential targets is beneficial to AD treatment.

Circular RNA (circRNA) is a noncoding RNA (ncRNA) formed by covalently closed-loop structure without 5′cap or a 3′ polyadenylated tail. By contrast to linear RNA, they are more stable in vivo due to their resistance to exonuclease degradation [2]. CircRNAs were found in tetrahymena, viruses and viroids decades ago, nevertheless, they were initially considered as by-products of splicing errors or mis-spliced RNAs [3]. Due to the advances in sequencing technology and bioinformatics analyses, circRNAs have been found to be specifically expressed in a cell type or developmental stage [4]. Recent studies show that circRNAs can serve as microRNA (miRNA) sponges to regulate gene expression [5]. There are growing evidence showing that circRNAs may have roles in the pathogenesis and diagnosis of disease via miRNA sponges. For an instance, circRNA-ZNF609 can sponge miR-150-5p to modulate AKT3 expression in Hirschsprung's disease [6]. Additionally, circRNA MTO1 functions as the sponge of mircoRNA-9 to suppress hepatocellular carcinoma progression [7]. Recent studies have shown that circRNAs may play roles in AD pathogenesis. For example, circRNA ciRS-7 has shown to bind miR-7 to degrade β-amyloid precursor protein (APP) and β-site APP cleaving enzyme 1 (BACE1), thus ciRS-7 may be a useful target in developing therapeutic strategies for AD [8]. Furthermore, our previous study has shown that 85 differentially expressed circRNAs were identified in AD model mice using circRNA microarray technology and among these aberrantly expressed circRNAs, mmu_circRNA_017963 has a high probability of participating in the biological processes involved in AD pathogenesis, suggesting that circRNAs may be potential therapeutic targets in AD [9]. Therefore, developing circRNA targeted therapeutic agents may provide a novel strategy for AD treatment. However, to date, no article has been reported in this field.

Panax notoginseng saponins (PNS) are the main active compound extracted from the root of Panax notoginseng, which is widely distributed in the Guangxi Zhuang Autonomous Region and Yunnan province. Study shows that there are about 20 saponin constituents contained in PNS and ginsenosides Rb1 and Rg1 are the main compounds in PNS [10]. Our previous studies showed that PNS could improve learning and memory performance in AD model mice senescence-accelerated mouse-prone 8 (SAMP8), alleviate the deposition of amyloid β-peptide (Aβ)1–40 and Aβ1–42 through inhibiting the expressions of *APP, BACE1* and *ADAM9* (a disintegrin and metalloprotease 9), increasing α-secretase activity and reducing β-secretase activity [11], and prevent oxidative stress injury via increasing the gene expressions and activities of SOD, CAT, and GSH-PX in the brains of SAMP8 [12], suggesting that PNS may be a promising candidate for AD treatment. However, the mechanisms by which PNS attenuates AD progression is still unclear. Since circRNAs play important roles in AD pathogenesis through regulating gene expression via miRNA sponges, we hypothesized that the therapeutic effects of PNS on AD might be via modulating the expression of AD associated circRNAs.

In the present study, we aimed to identify AD associated circRNAs profile in the hippocampus of SAMP8 after PNS treatment. After 3-month-old SAMP8 mice were intragastrically administrated PNS for 2 months, microarray technology was applied to analyze the differential expression profiles of circRNAs in the hippocampus of three PNS-treated and -untreated SAMP8 mice. The aberrantly expressed circRNAs were further validated by qRT-PCR and their miRNA binding sites were revealed, and the related mRNAs were predicted. This study is the first to identify circRNA profile in medicated AD model mice, and our data will provide useful resources for developing potential candidates targeting circRNAs against AD.

## 2 Materials and Methods

### 2.1 Animals, Drug Treatment and Preparation of Tissues

3-month-old SAMP8 were purchased from Tianjin University of Traditional Chinese Medicine (Tianjin, China). All the mice were pathogen- and virus-free. They were randomly divided into three groups: model group, PNS high-dosage, PNS low-dosage groups. Drug treatment was performed according to our previous study [12]. Briefly, the high- and low-dosage groups were intragastrically administrated 200 and 100 mg/kg of PNS (Yunke Pharmaceutical Manufacture Co., Ltd., Yunnan, China) every day, respectively, for 8 consecutive weeks. The same volume of distilled water was provided to the model group. The mice were ethically sacrificed and their hippocampal tissues were excised and stored at −80 °C prior to analysis. Animal care and experimental procedures were implemented according to the document “Guidance Suggestions for Caring for Laboratory Animals” produced by the Ministry of Science and Technology of China in 2006.

### 2.2 RNA Extraction and Reverse Transcription

Total RNA was extracted from each hippocampal tissue in TRIzol reagent (Invitrogen). For reverse transcription, 2 μg total RNA, 4 μL 5 × RT Buffer, 1 μL RT Enzyme Mix, 1 μL Primer Mix, and RNase free water were contained in the reaction system according to the instruction of ReverTra Ace qPCR RT Kit (Toyobo).

### 2.3 CircRNA Microarray

Three hippocampal tissues of model and PNS high-dosage group were used for microarray assay to measure differentially expressed circRNAs using the circRNAs chip (Arraystar mouse circRNAs chip, AraryStar) as indicated in our previous study [9].The microarray hybridization including purifying RNA, transcribing into fluorescent cRNA was performed based on the manufacturer's standard protocols and then hybridizing onto mouse circRNA arrays. After the hybridized slides were washed and fixed, the slides were scanned using Agilent Scanner G2505C, followed by the data collection by Agilent Feature Extraction software.

### 2.4 Microarray Analysis

The raw data were normalized using the Kangcheng homemade R software package (Kangcheng Bio-tech, Shanghai, China) as described in our previous study [9]. After prediction of miRNA targets of circRNAs and the circRNA/miRNA interaction based on miRWalk [13] and TargetScan [14], the miRNA support vector regression (mirSVR) algorithm was used to score and rank the efficiency of the predicted miRNA targets. 5 miRNAs with the highest mirSVR score were identified for the establishment of “Top5” circRNA-miRNA network [15].

### 2.5 qRT-PCR

Considering the fold change of expression tested in the microarray analysis and the predicted target miRNAs related with progression of AD in previous research, 9 differentially expressed circRNAs were selected for further investigation. The selected circRNAs were validated using qRT-PCR in triplicate in 15 samples of SAMP8 mice. Divergent primers of the selected circRNAs were designed and optimized according to the sequences of the circRNAs obtained from the database “circBase” (http://circbase.mdc-berlin.de). β-action (ACTB), a housekeeping gene, was used as a control. The primer sequences was summarized in Table 1. The appearance of a single peak in the melting curve of each sample demonstrated the specificity of the qRT-PCR results. The relative gene expression was calculated by 2 ^–ΔΔCp^ method, where ΔΔCp = ΔCp ^treatment^ − ΔCp ^control^ and ΔCp = Cp ^target gene^ − Cp^??CT?? gene^ [12,16].

**Table 1:** Primers designed for qRT-PCR validation of candidate circRNAs.

circRNAs	sense	anti-sense
mmu_circRNA_013636	5′−ATTTCAGGAAGTCACGAAGACC−3′	5′−TGGATGAGCCACAAACTGATT−3′
mmu_circRNA_013699	5′−AAAGCTCACCCAGGACCAGTT−3′	5′−TATGTTTCCTCGCTCAGTGCC−3′
mmu_circRNA_017963	5′−TGCGAGACAGCAGACGAAT−3′	5′−TTTGTATCAACTCCTGCATGAG−3′
mmu_circRNA_012180	5′−ACTGCACTCAAGGTCCAACA−3′	5′−CCATATTCTTCATAGCTGAGCA−3′
mmu_circRNA_006229	5′−GCATTTTACTGAAGGAGCCG−3′	5′−GAGGACCATGCTATTCTGGAAG−3′
mmu_circRNA_006173	5′−TCTACCAGGCTCTCCTCCCA−3′	5′−TCCAGGCACGTGCTCTGAG−3′
mmu_circRNA_003540	5′−TGACTGACGGCTCTGTTCTG −3′	5′−CAATTGTTACCTGCCCCATC −3′
mmu_circRNA_000692	5′−AATTCCTTCCAGTGGGCC −3′	5′−AGGTGGGCGTTATGGAAAT −3′
mmu_circRNA_001456	5′−CGCCGCCTCCTCCTCTCT−3′	5′−AGGACAAACCGGGGGTGAG −3′

### 2.6 Annotation and Function Prediction

According to the qRT-PCR results, mmu_circRNA_013636 and mmu_circRNA_012180 were selected for annotation and function prediction based on circRNA-miRNA-gene network in the light of the analysis of miRWalk (http://zmf.umm.uni-heidelberg.de/apps/zmf/mirwalk2/), TargetScan (http://www.targetscan.org/) and miRanda (www.microrna.org/). In addition, cytoscape (http://www.cytoscape. org/) was utilized to establish a circRNA-miRNA-mRNA interaction network of the selected circRNAs. Functional annotation of genes in the networks were performed using KEGG and GO pathway analysis.

### 2.7 Statistical Analysis

Analysis of variance was performed with SPSS software for windows 13.0. The significance of qRT-PCR validation between each groups was tested by one-way analysis of variance (ANOVA), and P < 0.05 was considered statistically significant.

## 3 Results

### 3.1 CircRNA Expression Profile in Hippocampal Tissues of PNS-Treated Relative to PNS-Untreated SAMP8

**Fig. 1:**
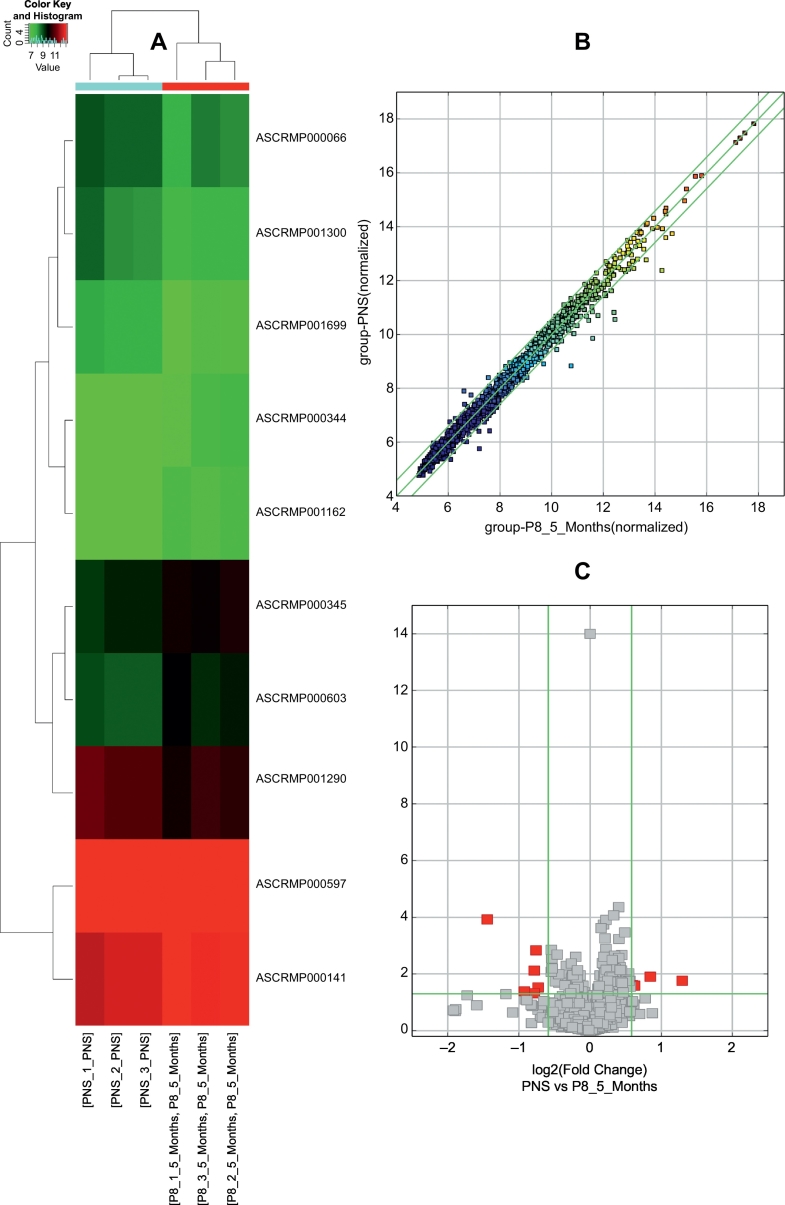
The hierarchical cluster, scatter plot and volcano plot of differential expression of circRNAs in PNS-treated and –untreated SAMP8 mice. A: Hierarchical cluster of differentially expressed circRNAs. “Green” indicates low intensity, “black” indicates medium intensity and “red” indicates strong intensity. B: Scatter plot of circRNA signal values. The values of X and Y axes represents the normalized signal values of the samples (log2 scaled) and the averaged normalized signal values of samples (log2 scaled) respectively. The green lines are fold change lines. The CircRNAs above the top green line and below the bottom green line demonstrates >1.5-fold change of circRNAs between the two compared samples. C: Volcano plot of differential expression of circRNAs. The vertical lines correspond to 1.5-fold up and down, respectively. The horizontal line represents a P-value of 0.05, and the red point in the plot represents the differentially expressed circRNAs with statistical significance.

The results of circRNA microarray showed that 10 circRNAs were differentially expressed in hippocampal tissues of PNS-treated and –untreated SAMP8 (fold change>1.5; P < 0.05). Among these circRNAs, four circRNAs were upregulated and six circRNAs were downregulated in PNS-untreated samples compared to PNS-treated samples. As demonstrated in Fig. 1A, the expression patterns of circRNAs in hippocampal tissues of PNS-treated and –untreated SAMP8 were classified by unsupervised hierarchical clustering. Fig. 1B showed a scatter plot, which depicts the variation in circRNA expression between PNS-treated and –untreated SAMP8 hippocampal samples. A volcano plot visualizing the statistically significant difference of circRNAs between two groups was showed in Fig. 1C.

### 3.2 Validation of Aberrantly Expressed CircRNA Using Quantitative Real-Time Polymerase Chain Reaction (qRT-PCR)

**Fig. 2:**
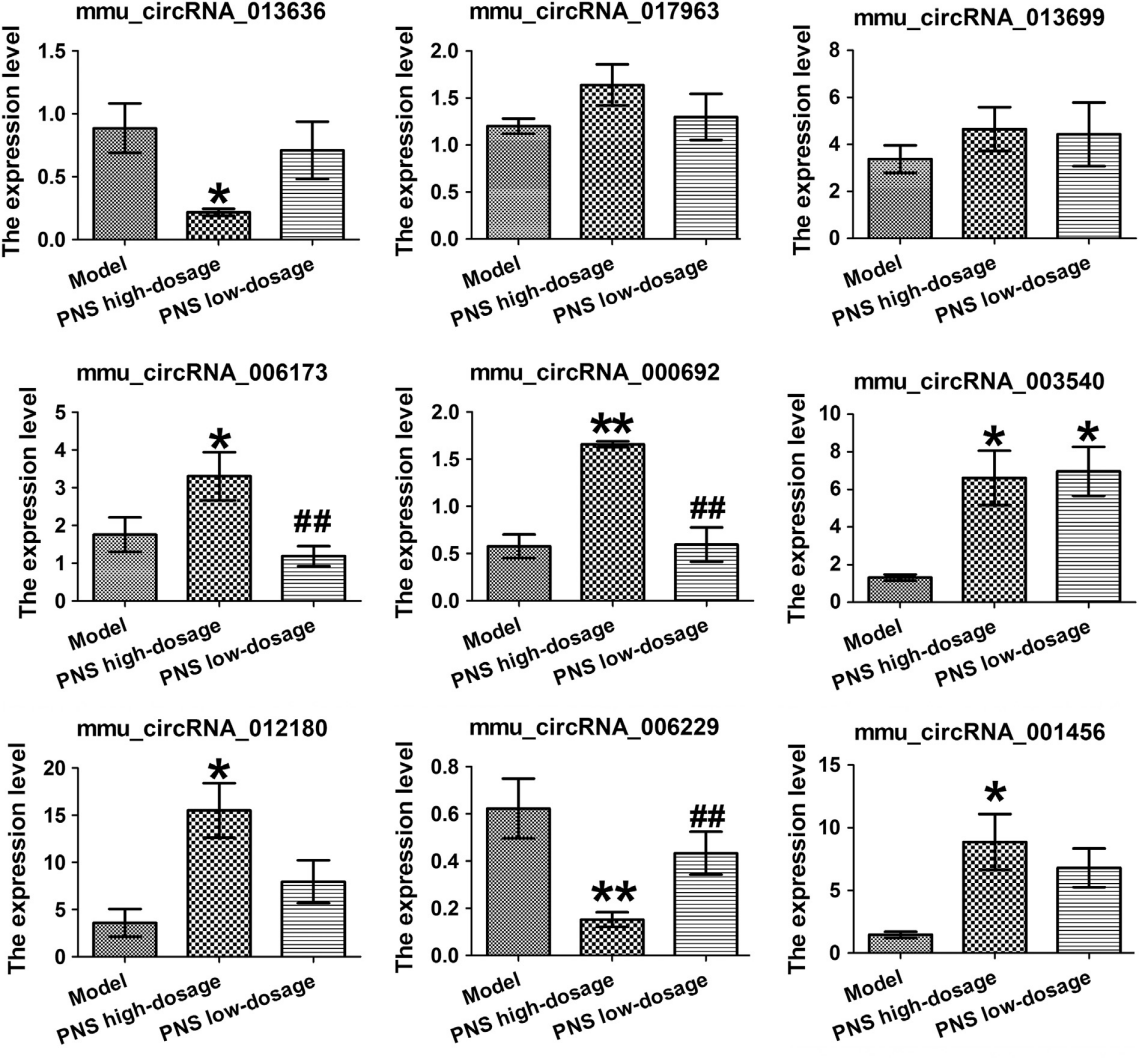
The expression levels of candidate circRNAs for validation by qRT-PCR in 15 SAMP8 hippocampal tissues. Statistically differences were calculated by one-way ANOVA using SPSS 13.0 software. *P < 0.05, **P < 0.01 versus SAMP8 group.

The false discovery rate (FDR) method were to adjust P values since false positives may occur due to multi-comparisons. After FDR correction, 9 differentially expressed circRNAs were selected to validate the accuracy and reliability using qRT-PCR analysis. The results showed that 7 (mmu_circRNA_006173, mmu_circRNA_001456, mmu circRNA_006229 mmu_circRNA_003540, mmu_circRNA_012180, mmu_circRNA_000692 and mmu_circRNA_013636) out of the 9 circRNAs remarkably differed between the hippocampal tissues of PNS-treated and-untreated SAMP8 (P < 0.05) (Fig. 2).

### 3.3 Annotation and Prediction of mmu_circRNA_013636 and mmu_circRNA_012180 Targeted miRNA-mRNA Network

**Fig. 3:**
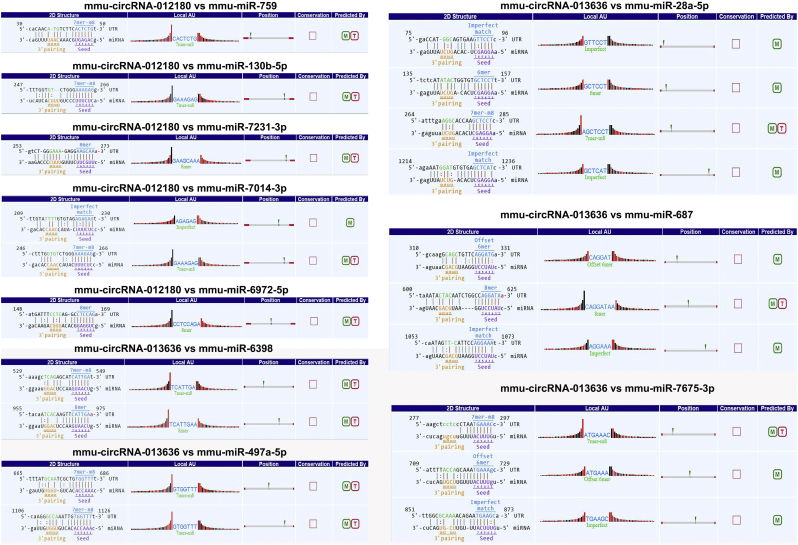
Five detailed annotation for the circRNA/miRNA interaction (mmu_circRNA_013636 and mmu_circRNA_012180 and their Top-5 predicted miRNA targets).

Theoretically, the interactions between circRNAs and their miRNA targets could be predicted by conserved seed-matching sequences. Thus the functions of circRNAs could be predicted according to their target miRNAs. For determining the potential miRNAs, two confirmed circRNAs (mmu_circRNA_013636, best transcript: *Trpc6*, chr9:8634046–8,658,377; mmu_circRNA_012180, best transcript: *Phkb*, chr8:88420328–88,446,141) were selected, and five highest-ranking miRNA binding targets (“Top 5”) were confirmed based on mirSVR scores. Fig. 3 illustrated the molecular interactions between circRNAs and their Top-5 miRNA targets by their specific base pairing. We hypothesized that circRNAs could act as miRNA sponges to regulate the activity of targeted miRNAs and thus to modulate circRNA-miRNA-mRNA network, and that the interaction network could be predicted via miRanda [17], TargetScan [14] and miRWalk [18]. In the light of the above analysis tools, we predicted a total of 442 and 631 potential mRNAs to interact with mmu_circRNA_013636 and mmu_circRNA_012180 respectively.

**Fig. 4:**
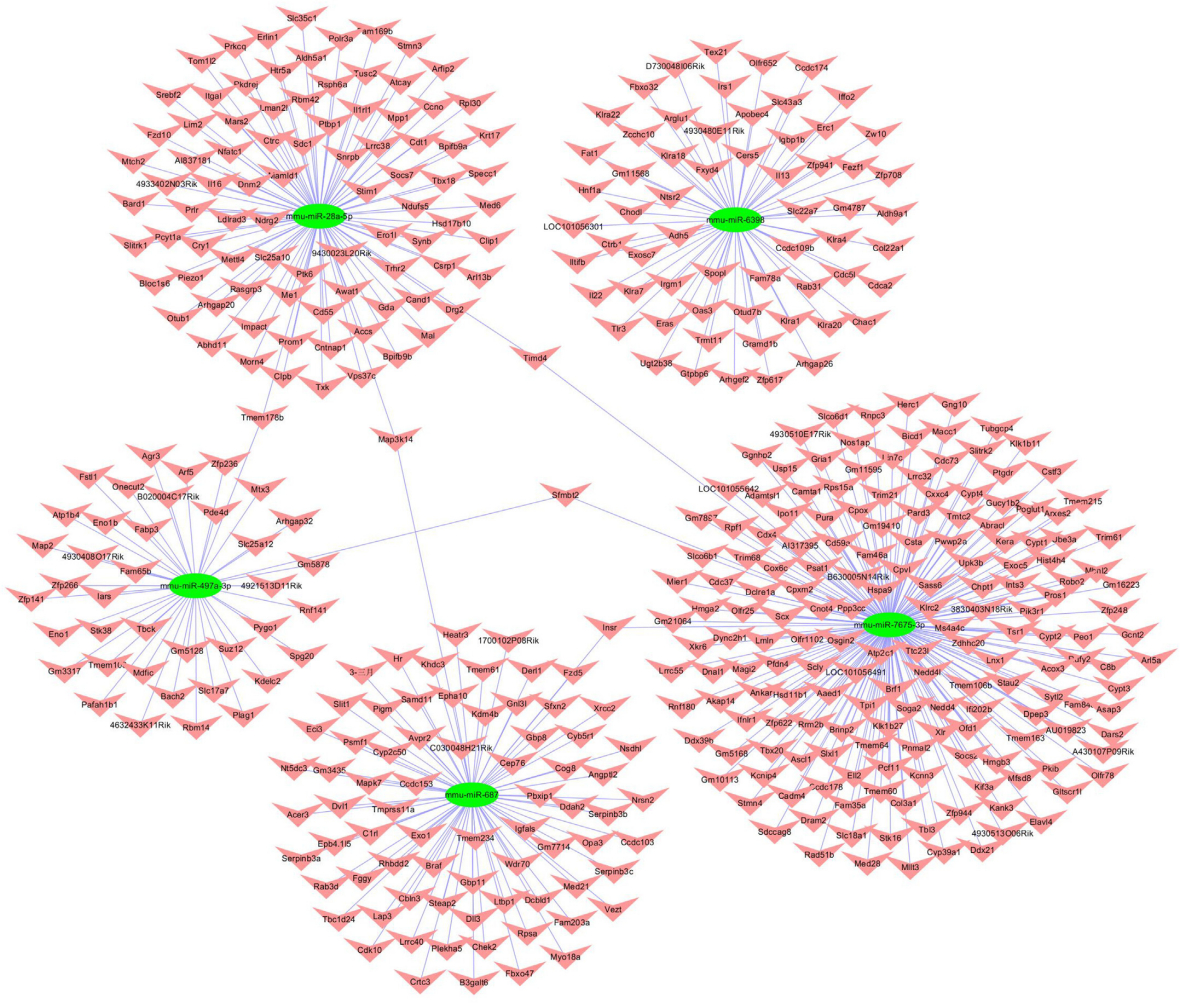
The predicted mmu_circRNA_013636 targeted circRNA-miRNA-mRNA interaction network according to sequence-pairing prediction. The miRNA-binding sites were predicted by mirSVR, and targeted miRNAs and mRNAs were predicted by miRWalk and TargetScan. Five miRNAs were observed with overlapping results.

**Fig. 5:**
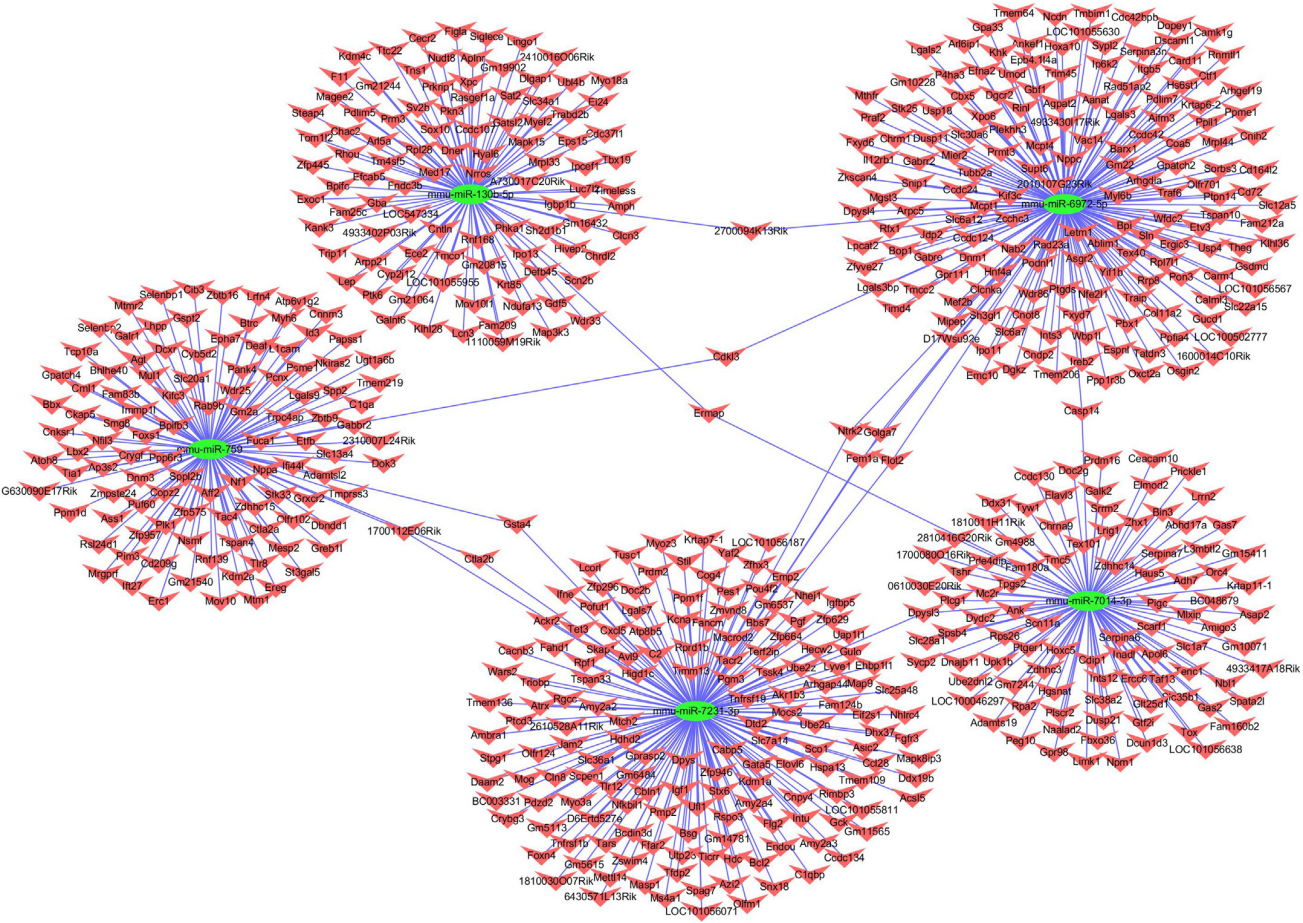
The predicted mmu_circRNA_012180 targeted circRNA-miRNA-mRNA interaction network according to sequence-pairing prediction. The miRNA-binding sites were predicted by mirSVR, and targeted miRNAs and mRNAs were predicted by miRWalk and TargetScan. Five miRNAs were observed with overlapping results.

**Fig. 6:**
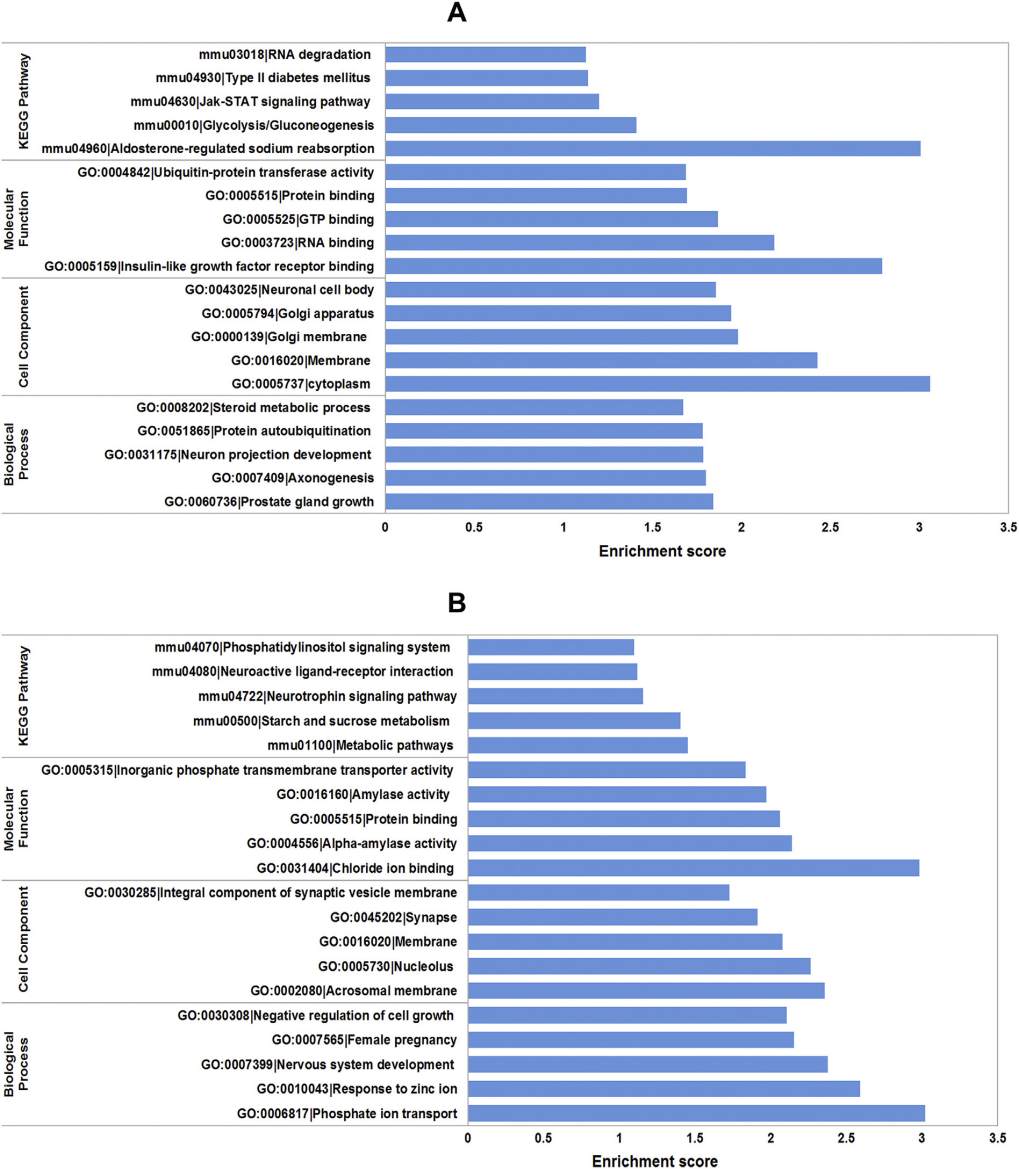
GO analysis according to circRNA-miRNAs-mRNAs network. (A) mmu_circRNA_013636; (B) mmu_circRNA_012180. The x- and y-axis represent the top 5 significantly enriched biological processes, cell component and molecular function and their scores (−log10 (P value)), respectively. The horizontal axis represents the significant level of GOs and KEGG pathways.

Moreover, cytoscape software was utilized to construct the circRNA-miRNA-mRNA interaction network of mmu_circRNA_013636 and mmu_circRNA_012180. As shown in Fig. 4, Fig. 5, mmu_miR_7675-3p and mmu_miR_7231-3p showed the largest interaction network of mmu_circRNA_013636 and mmu_circRNA_012180 respectively. The KEGG and GO pathway analyses were performed to analyze the function of mmu_circRNA_013636 and mmu_circRNA_012180 based on the results of miRanda, TargetScan and miRWalk. As illustrated in Fig. 6A and B, mmu_circRNA_013636 was strongly related with the regulation of prostate gland growth (biological process, GO: 0060736), steroid metabolic process (biological process, GO: 0008202), protein autoubiquitination (biological process, GO: 0051865), neuron projection development (biological process, GO: 0031175) and axonogenesis (biological process, GO: 0007409). Meanwhile, mmu_circRNA_012180 was associated with phosphate ion transport (biological process, GO: 0006818), response to zinc ion (biological process, GO: 0010043), nervous system development (biological process, GO: 0007399), female pregnancy (biological process, GO: 0007565) and negative regulation of cell growth (biological process, GO: 0030308). Additionally, “Top 5”pathways highly associated to the mRNAs predicted by the targeted miRNAs of mmu_circRNA_013636 and mmu_circRNA_012180 were predicted using KEGG enrichment analysis and illustrated in Fig. 6 A and B.

## 4 Discussion

Since circRNAs play important roles in AD pathogenesis, they are considered as potential therapeutic targets in AD. Thus, developing therapeutic candidates targeting circRNAs may provide a novel therapeutic strategy for AD treatment. However, very little is known about the research of circRNA-targeted agents. The present study uniquely shows that PNS may exhibit an ability to modulate the expression of AD-associated circRNAs. Specifically, PNS treatment leads to 10 differentially expressed circRNAs (including four upregulated and six downregulated circRNAs) in the hippocampus tissues of SAMP8. Among these aberrantly expressed circRNAs, 9 circRNAs were selected and 5 circRNAs including mmu_circRNA_006173, mmu_ circRNA_001456, mmu_circRNA_003540, mmu_circRNA_012180 and mmu_circRNA_000692 were validated to be remarkably downregulated in the hippocampal tissues of PNS-untreated SAMP8 as compared to PNS-treated samples, and mmu_circRNA_006229, mmu_circRNA_ 013636 were significantly upregulated in PNS-untreated samples compared to PNS-treated samples (Fig. 2). These results demonstrate that the therapeutic effect of PNS against AD may be associated with its role in the regulation of circRNA expression. In conclusion, this work may provide a novel thought for developing potential agents against AD.

CircRNAs can function as miRNA sponge via their miRNA binding sites. It was found that circRNAs have more miRNA binding sites as compared to linear miRNA sponges [5]. Thus, via circRNA-targeted miRNA-mediated impacts on gene expression, circRNAs might play important roles in AD pathogenesis. In the present study, we observed that “Top5” miRNAs that might potentially interact with mmu_circRNA_013636 and mmu_circRNA_012180 were confirmed based on mirSVR scores (Fig. 3). Since the relationship between these sponged miRNAs and AD remains largely unknown currently, cytoscape was utilized to identify the function of mmu_circRNA_013636 and mmu_circRNA_012180 by analyzing the circRNA-miRNA-mRNA interaction network. The results showed that mmu_miR_7675-3p and mmu_miR_7231-3p showed the largest interaction network of mmu_circRNA_013636 and mmu_circRNA_ 012180 respectively (Fig. 4, Fig. 5). In addition, using GO and KEGG pathway analyses, the prediction of potential functions of mmu_circRNA_013636 and mmu_circRNA_012180 was performed by examining miRNA-targeted genes. The results of GO analysis showed that mmu_circRNA_013636 was possibly participating in the biological processes of prostate gland growth, steroid metabolic process, protein autoubiquitination, neuron projection development and axonogenesis. Except for prostate gland growth, the other four biological processes have been reported to play important roles in AD pathogenesis [[19], [20], [21], [22]]. Additionally, mmu_circRNA_012180 might be associated with the biological processes of phosphate ion transport, response to zinc ion, nervous system development, female pregnancy and negative regulation of cell growth. Among these biological processes, the processes of phosphate ion transport, response to zinc ion and negative regulation of cell growth were validated to have a role in AD development [[23], [24], [25]]. KEGG pathway analysis revealed that mmu_circRNA_013636 has a high possibility to participate in the pathways of RNA degradation, type II diabetes mellitus, JAK-STAT, glycolysis/gluconeogenesis and aldosterone-regulated sodium reabsorption (Fig. 6 A and B). Among these signaling pathways, RNA degradation, type II diabetes mellitus, JAK-STAT and glycolysis/gluconeogenesis signaling pathways have been proven to contribute to AD pathogenesis [[26], [27], [28], [29]]. Meanwhile, mmu_circRNA_012180 is possibly involved in metabolic, neurotrophin, starch and sucrose metabolism and phosphatidylinositol signaling pathways known to potentially mediated AD pathogenesis [[30], [31], [32], [33]] (Fig. 6 A and B). Consequently, in consideration of these results, we hypnotized that mmu_circRNA_013636 and mmu_circRNA_012180 may be involved in AD-associated signaling pathways. However, the potential role of mmu_circRNA_013636 and mmu_circRNA_012180 in AD pathogenesis remains unclear. Thus, the function of mmu_circRNA_017963 should be investigated in further study.

Some limitations in our results should be interpreted. Firstly, when evaluating the expression of circRNAs, false negatives is inevitable since the minimum detection thresholds is relatively low due to the low level of circRNAs. Secondly, the circRNA-targeted miRNAs and mRNAs should be experimentally identified in further study. Thirdly, since circRNAs are mostly derived from precursor mRNA via exon circularization [2,4], the effect of PNS on the expression of circRNA-derived mRNA should be investigated in the future. Fourthly, gain and loss of function assays should be performed to confirm the roles of circRNAs in AD pathogenies and the contribution of circRNAs to the effect of PNS against AD in future study. Finally, RNA-sequence technology should be performed to uncover new transcriptome complexities since circRNA expression microarrays used in the study lack the ability to identify novel features of transcriptome.

## 5 Conclusion

Collectively, our study indicates that the therapeutic effects of PNS on AD may be through modulating the expression of AD associated circRNAs and suggests that PNS is a potential circRNA-targeted agent against AD, which may provide useful resources for developing potential candidates targeting circRNAs against AD.

## Conflicts of Interest

The authors have no conflicts of interest to disclose.

## Appendix A Supplementary data

Supplementary material 1 Image 1

Supplementary material 2 Image 2
