# Supplementary material for: Identification of Differentially Expressed Profiles of Alzheimer's Disease Associated Circular RNAs in a Panax Notoginseng Saponins-Treated Alzheimer's Disease Mouse Model
Source: Comput Struct Biotechnol J. 2018 Nov 14;16:523–31. doi: 10.1016/j.csbj.2018.10.010 (PMC6260282; doi:10.1016/j.csbj.2018.10.010)
Supplement: Supplementary file 1 — Supplementary material 1 [file mmc1.docx]

| circRNAs | p-value | FDR | Fold change | Regulation | chrom | strand | circRNA_type | Gene symbol |
| --- | --- | --- | --- | --- | --- | --- | --- | --- |
| mmu_circRNA_013636 | 1.52796E-05 | 0.002291946 | 37.7211457 | down | chr9 | + | exonic | Trpc6 |
| mmu_circRNA_013699 | 0.000795782 | 0.020200617 | 2.1175951 | up | chr10 | - | exonic | Stxbp5 |
| mmu_circRNA_017963 | 5.38477E-06 | 0.001533551 | 6.4336662 | down | chr10 | - | exonic | Tbc1d30 |
| mmu_circRNA_012180 | 0.000117474 | 0.040141177 | 2.7239694 | down | chr8 | + | exonic | Phkb |
| mmu_circRNA_006229 | 0.007485974 | 0.0279670259 | 1.7218934 | down | chr12 | - | exonic | Pigh |
| mmu_circRNA_006173 | 0.040592219 | 0.042362084 | 1.8951038 | up | chr12 | - | intragenic | Max |
| mmu_circRNA_003540 | 0.001455215 | 0.0184700334 | 1.6912061 | down | chr3 | + | exonic | Usp13 |
| mmu_circRNA_000692 | 0.00028805 | 0.013579517 | 2.9873557 | up | chr9 | - | exonic | Ube2cbp |
| mmu_circRNA_001456 | 0.029445448 | 0.0354634955 | 1.6598096 | down | chr17 | + | intragenic | Rn45s |
| mmu_circRNA_003539 | 0.02458409 | 0.335812178 | 1.5068962 | up | chr8 | - | exonic | Csmd1 |

Supplementary table. 1 Differently expressed circRNAs in microarray analysis
